# Risk factors of clinically relevant postoperative pancreatic fistula after pancreaticoduodenectomy: A systematic review and meta-analysis

**DOI:** 10.1097/MD.0000000000029757

**Published:** 2022-06-30

**Authors:** Biao Zhang, Qihang Yuan, Shuang Li, Zhaohui Xu, Xu Chen, Lunxu Li, Dong Shang

**Affiliations:** a Department of General Surgery, Clinical Laboratory of Integrative Medicine, The First Affiliated Hospital of Dalian Medical University, Dalian, Liaoning, China; b Institute of Integrative Medicine, Dalian Medical University, Dalian, Liaoning, China; c Department of Hernia and Colorectal Surgery, The Second Affiliated Hospital of Dalian Medical University, Dalian, Liaoning, China.

**Keywords:** clinically relevant pancreatic fistula, meta-analysis, pancreaticoduodenectomy, risk factor, systematic review

## Abstract

Clinically relevant postoperative pancreatic fistula (CR-POPF) is a common and troublesome complication after pancreatoduodenectomy (PD). We conducted a systematic review and meta-analysis to identify the risk factors of CR-POPF after PD.

**Methods:**

We searched PubMed, EMBASE, and Cochrane Library databases for studies related to risk factors of CR-POPF after PD. Odds ratios (ORs) with corresponding 95% confidence intervals (CIs) were extracted from the included studies, then a meta-analysis was conducted. If necessary, sensitivity analysis would be performed by changing the effect model or excluding 1 study at a time. Publication bias was assessed by funnel plot and Begg test and Egger test.

**Results:**

A total of 27 studies with 24,740 patients were included, and CR-POPF occurred in 3843 patients (incidence = 17%, 95% CI: 16%–19%). Male (OR = 1.56, 95% CI: 1.42–1.70), body mass index >25 kg/m^2^ (OR = 1.98, 95% CI: 1.23–3.18), pancreatic duct diameter <3 mm (OR = 1.87, 95% CI: 1.66–2.12), soft pancreatic texture (OR = 3.49, 95% CI: 2.61–4.67), and blood transfusion (OR = 3.10, 95% CI: 2.01–4.77) can significantly increase the risk of CR-POPF. Pancreatic adenocarcinoma (OR = 0.54, 95% CI: 0.47–0.61), vascular resection (OR = 0.57, 95% CI: 0.39–0.83), and preoperative chemoradiotherapy (OR = 0.68, 95% CI: 0.57–0.81) can significantly decrease the factor of CR-POPF. Diabetes mellitus was not statistically associated with CR-POPF (OR = 0.66, 95% CI: 0.40–1.08). However, the analysis of body mass index, pancreatic texture, and diabetes mellitus had a high heterogeneity, then sensitivity analysis was performed, and the result after sensitivity analysis showed diabetes mellitus can significantly decrease the risk of CR-POPF. There was no significant publication bias in this meta-analysis.

**Conclusions:**

The current review assessed the effects of different factors on CR-POPF. This can provide a basis for the prevention and management of CR-POPF. Effective interventions targeting the above risk factors should be investigated in future studies for decreasing the occurrence of CR-POPF.

## 1. Introduction

Pancreatoduodenectomy (PD) is a classic surgery for tumors originating from the pancreatic head, lower common bile duct, and periampullary region.^[1]^ PD has always been a challenging surgery with high mortality and morbidity. Although the mortality of PD has reduced to about 2%, the postoperative morbidity is as high as 30% to 50%. Pancreatic fistula (PF) is one of the most important complications after PD, with an incidence of about 10% to 28%.^[2]^ According to the definition of the International Study Group on Pancreatic Fistula (ISGPF) in 2016, grade A PF was replaced by biochemical leak because of no clinical impact, and grades B and C PF are also named as clinically relevant postoperative PF (CR-POPF).^[3]^ Compared with grade A PF, CR-POPF not only can increase the length and cost of hospitalization, but can also increase the risk of other morbidities such as postoperative bleeding, abdominal infection, and even death.^[4–6]^ Therefore, it has an essential significance to prevent the occurrence of CR-POPF after PD. Identification of risk factors for CR-POPF can better understand the potential mechanisms of CR-POPF and is also conducive to the prevention and management of CR-POPF. Many studies have extensively investigated the risk factors of PF, such as increased body mass index (BMI), smaller pancreatic duct diameter, soft pancreatic texture, lower serum albumin, surgical techniques, etc.^[7–9]^ However, most previous studies related to risk factors of PF did not exclude grade A PF, and the effects of different factors on CR-POPF were still unclear. In the current article, we conducted a systematic review and meta-analysis to identify the risk factors for CR-POPF after PD, thereby providing a basis for the prevention and management of CR-POPF.

## 2. Methods

Our protocol was registered in the International Prospective Register of Systematic Reviews on December 9, 2020 (PROSPERO CRD42020219814). This systematic review and meta-analysis was performed according to the Preferred Reporting Items for Systematic Reviews and Meta-Analysis guidelines.^[10]^

### 2.1. Search strategy

Relevant studies were searched from PubMed, EMBASE, and Cochrane Library databases from inception to November 2019 with no limitation of language. We used the following combined Medical Subject Heading terms which included “risk factors”, “pancreatic fistula”, and “pancreaticoduodenectomy”. Furthermore, we also scanned the references from included studies in order not to miss relevant studies.

### 2.2. Eligibility criteria

Studies that met all the following conditions were considered for eligibility: patients underwent PD; studies analyzed the risk factors of CR-POPF; the diagnosis of CR-POPF met the definition of ISGPF; studies provided the result of multivariate logistic regression analysis, odds ratio (OR) with corresponding 95% confidence interval (CI), *P* values, or relevant values could be calculated based on the original data reported in the study; retrospective or prospective studies.

Studies with duplicate data were excluded.

### 2.3. Data extraction

Two researchers independently conducted data extraction according to a standardized data collection form, and the disagreement would be resolved by discussion. The extracted data included first author, publication year, nation, source of patients, recruitment period, study design, number of patients, number of CR-POPF, risk factors, and OR with corresponding 95% CI.

### 2.4. Quality assessment

The quality of the included studies was assessed by 2 researchers according to the Newcastle-Ottawa Scale (NOS).^[11]^ The NOS contains 3 aspects and 8 items: selection (4 items, 1 star each); comparability (1 item, up to 2 stars); and outcome (3 items, 1 star each) for a cohort study or exposure (3 items, 1 star each) for case–control study. The score of NOS is on a scale of between 0 and 9 stars, and the study with NOS score >6 stars was considered as a high-quality study.^[12]^

### 2.5. Data synthesis and analysis

We used Review Manager software (version 5.3; The Cochrane Collaboration, Copenhagen, Denmark) and Stata statistical software (version 14.0) for the statistical processing. The pooled OR with corresponding 95% CI was calculated, and *P* value of <.05 was considered statistically significant. Heterogeneity was assessed by Cochran’s Q test and the inconsistency (*I*^2^) statistic. *I*^2^ <50% represented a low heterogeneity, and the fixed effects model would be used. If not, the random effects model would be used. Sensitivity analysis was performed through changing the effect model or excluding 1 study at a time. If >10 studies were included, publication bias would be assessed by funnel plot and Begg and Egger tests, and *P* value of <.05 indicated significant publication bias.

## 3. Results

### 3.1. Search result

**Figure 1.**
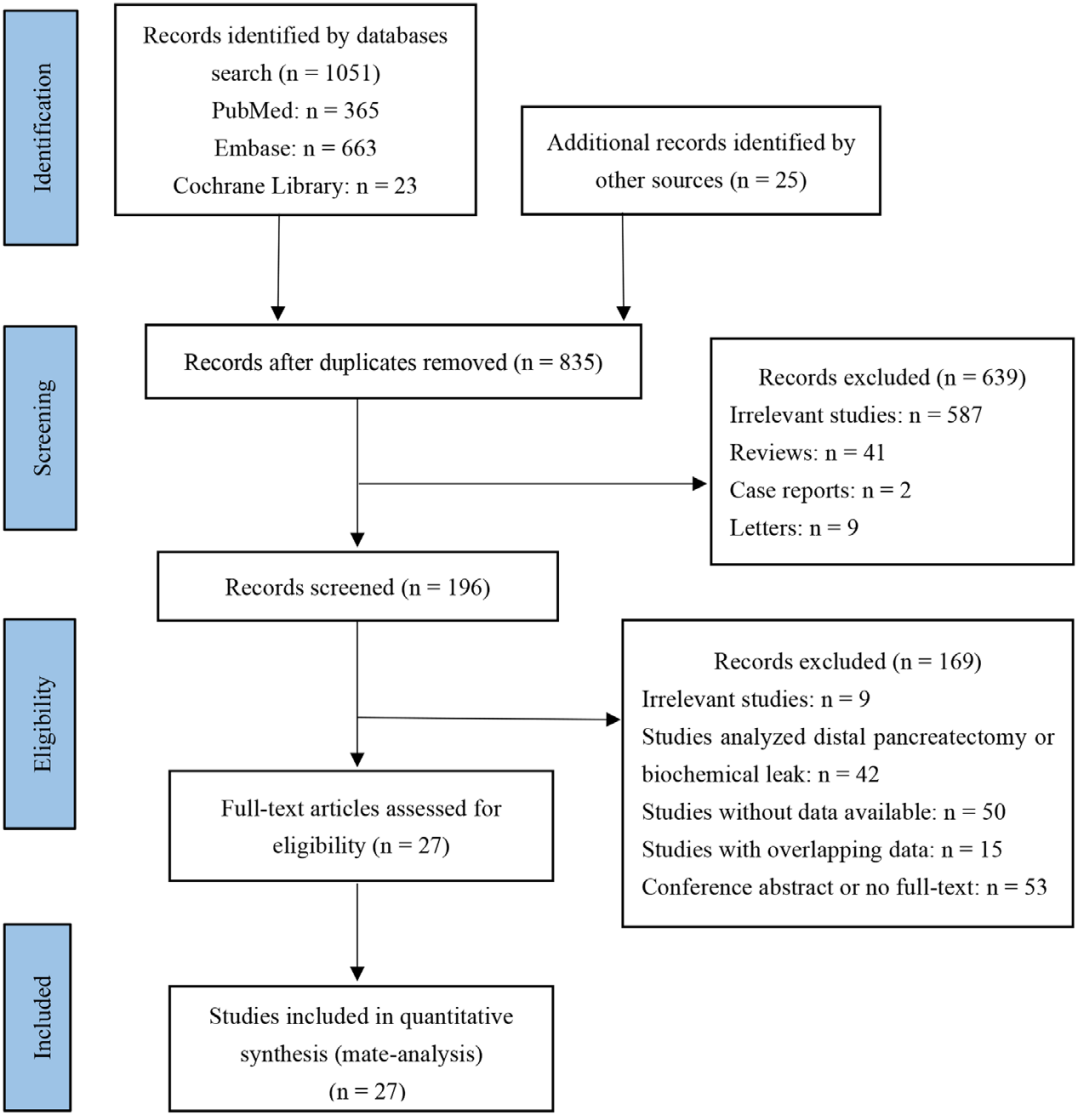
Flow diagram of study inclusion and exclusion.

One thousand seventy-six studies were acquired by initial search, then 241 duplicate studies were excluded, 639 studies were excluded by scanning title and abstract, 169 studies were excluded by reading full text, and finally 27 studies^[13–39]^ were included. The process of study exclusion and inclusion is shown in Figure 1. Of the 27 included studies, 3 studies were from the United States, 2 studies were from Italy, 3 studies were from France, 1 study was from Sweden, 6 studies were from China, 11 studies were from Japan, and 1 study was from Korea. Three studies were prospective and 24 studies were retrospective. The NOS scores of 27 studies were between 7 and 9 stars. The characteristics of the 27 included studies are shown in Table 1.

**Table 1:** Characteristics of the included studies.

Study	Nation	Study period	Study design	Patients number	Number of CR-POPF	Score of NOS	Risk factors
Pratt et al^[13]^ (2008)	USA	2001.10–2007.03	Prospective	233	31	8	4. 5
Kawai et al^[14]^ (2011)	Japan	2005.07–2009.06	Retrospective	1239	178	8	1. 4
Hiyoshi et al^[15]^ (2013)	Japan	2002.03–2010.10	Retrospective	176	30	8	1
Malleo et al^[16]^ (2013)	Italy	2000.01–2011.12	Prospective	602	63	8	1. 3. 4. 6
Kanda et al^[17]^ (2014)	Japan	2008.01–2013.03	Retrospective	246	57	7	4
Chen et al^[18]^ (2015)	China	2008.01–2013.12	Retrospective	921	89	8	4. 7
Fujiwara et al^[19]^ (2015)	Japan	2000.05–2013.05	Retrospective	247	43	8	1
Nagai et al^[20]^ (2015)	Japan	2007–2013	Retrospective	254	44	9	4. 8. 9
Nishida et al^[21]^ (2016)	Japan	2010.01–2014.12	Retrospective	266	43	8	2. 4. 5. 8
Yamashita et al^[22]^ (2016)	Japan	2007.01–2012.12	Retrospective	174	18	8	1. 2. 6
Kantor et al^[23]^ (2017)	USA	2011–2012	Retrospective	1731	270	8	1. 2
Partelli et al^[24]^ (2017)	Italy	2013–2015	Retrospective	463	64	8	1. 7
Xia et al^[25]^ (2018)	China	2009.01–2015.12	Retrospective	225	40	9	4
Ke et al^[26]^ (2018)	China	2015.09–2017.08	Retrospective	170	44	9	4. 6
Ellis et al^[27]^ (2019)	USA	2014–2017	Retrospective	10,022	1658	8	1. 3. 5. 9
Kang et al^[28]^ (2019)	Korea	2007–2014	Prospective	1898	275	9	1. 4. 5. 6. 8
Li et al^[29]^ (2019)	China	2011.01–2016.12	Retrospective	189	38	7	3. 4
Zarzavadjian Le Bian et al^[30]^ (2019)	France	2004.02–2016.12	Retrospective	270	74	8	2. 7
Abe et al^[31]^ (2020)	Japan	2006–2018	Retrospective	136	37	9	1
Bardol et al^[32]^ (2020)	France	2009.01–2018.04	Retrospective	195	58	8	3. 4. 5. 8. 9
Ohgi et al^[33]^ (2020)	Japan	2010.01–2014.12	Retrospective	346	116	8	2. 3. 4. 5. 8
Tabchouri et al^[34]^ (2020)	France	2012–2017	Retrospective	448	103	8	1. 4. 5. 9
Utsumi et al^[35]^ (2020)	Japan	2008.04–2018.09	Retrospective	108	32	7	5
Williamsson et al^[36]^ (2020)	Sweden	2010.01–2018.06	Retrospective	2503	245	8	1. 6. 8
Zou et al^[37]^ (2020)	China	2005.01–2016.12	Retrospective	707	75	7	2
Huang et al^[38]^ (2020)	China	2010.01–2018.05	Retrospective	762	82	8	3. 4. 5
Nakamura et al^[39]^ (2020)	Japan	2013.05–2018.05	Retrospective	209	36	8	3. 4. 5. 9

Remarks = 1: gender, 2: BMI (25 kg/m^2^), 3: pancreatic duct diameter (3 mm), 4: pancreatic texture, 5: pathology type, 6: diabetes mellitus, 7: Blood transfusion, 8: vascular resection, 9: preoperative chemoradiotherapy.

BMI = body mass index, CR-POPF = clinically relevant postoperative pancreatic fistula, NOS = Newcastle-Ottawa Scale.

### 3.2. Risk factors of CR-POPF

**Figure 2.**
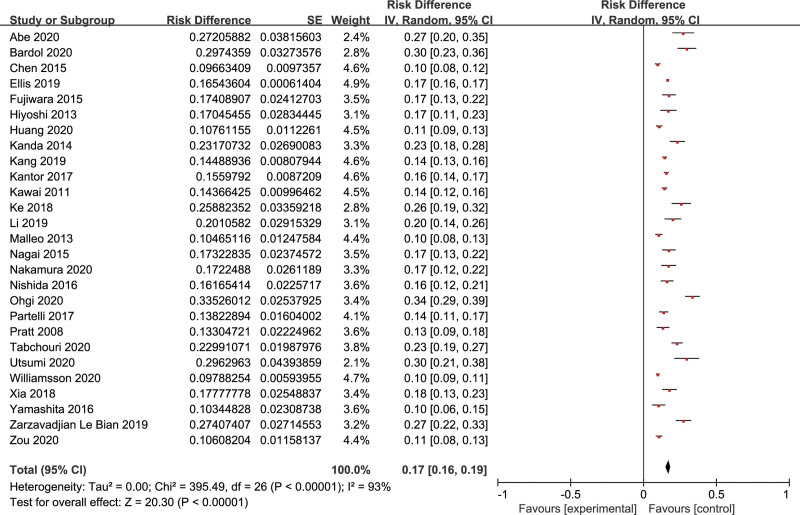
The pooled incidence of CR-POPF after PD. CR-POPF = clinically relevant postoperative pancreatic fistula, PD = pancreatoduodenectomy.

Twenty-seven studies included 24,740 patients who underwent PD, and CR-POPF occurred in 3843 patients; the pooled incidence was 17% (95% CI: 16%–19%) (Fig. 2). Nine factors had sufficient data and were reported in at least 3 studies. A meta-analysis was conducted for these factors: gender, BMI (25 kg/m^2^), pancreatic duct diameter (3 mm), pancreatic texture, pathology type, diabetes mellitus, blood transfusion, vascular resection, and preoperative chemoradiotherapy. And the meta-analysis results of risk factors are summarized in Table 2.

**Table 2:** The summarized results of risk factors.

Risk factors	Number of studies	OR	95% CI	*P*	*I* ^2^
Gender	12	1.56	1.42–1.70	<.001	29%
BMI (25 kg/m^2^)	6	1.98	1.23–3.18	<.001	80%
Pancreatic duct diameter (3 mm)	7	1.87	1.66–2.12	<.001	28%
Pancreatic texture	16	3.49	2.61–4.67	<.001	57%
Pathology type	10	0.54	0.47–0.61	<.001	33%
Diabetes mellitus	5	0.66	0.40–1.08	0.10	62%
Blood transfusion	3	3.10	2.01–4.77	<.001	0%
Vascular resection	6	0.57	0.39–0.83	.003	0%
Preoperative chemoradiotherapy	5	0.68	0.57–0.81	<.001	49%

BMI = body mass index, CI = confidence interval, OR = odds ratio.

#### 3.2.1. Gender

**Figure 3.**
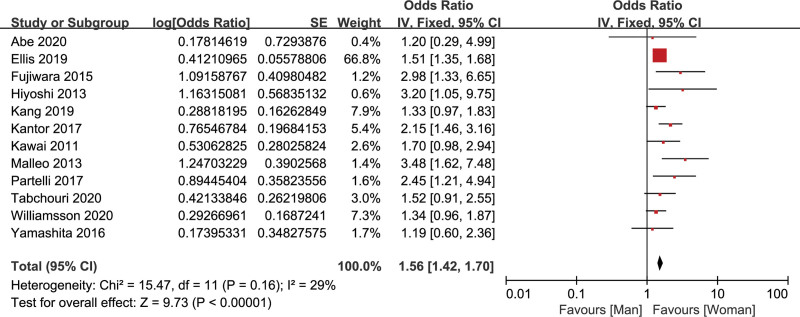
Forest plot of the relationship between CR-POPF and gender. CR-POPF = clinically relevant postoperative pancreatic fistula.

Twelve studies^[14–16,19,22–24,27,28,31,34,36]^ analyzed the relationship of gender and CR-POPF, containing 19,636 patients. There was a low heterogeneity (*I*^2^ = 29%), so the fixed effects model was used for data synthesis. The pooled data showed that male was a significant risk factor of CR-POPF (OR = 1.56, 95% CI: 1.42–1.70, *P* < .001) (Fig. 3).

#### 3.2.2. Body mass index (25 kg/m^2^)

**Figure 4.**
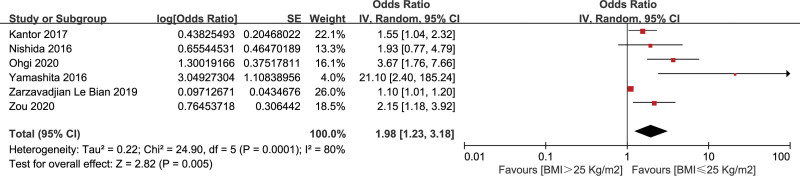
Forest plot of the relationship between CR-POPF and BMI (25 kg/m^2^). BMI = body mass index, CR-POPF = clinically relevant postoperative pancreatic fistula.

Six studies^[21–23,30,33,37]^ reported the effect of BMI >25 kg/m^2^ on CR-POPF, including 3494 patients. There was a high heterogeneity (*I*^2^ = 80%), so the random effects model was adopted. The result indicated that BMI >25 kg/m^2^ was a significant risk factor of CR-POPF (OR = 1.98, 95% CI: 1.23–3.18, *P* < .001) (Fig. 4).

#### 3.2.3. Pancreatic duct diameter (3 mm)

**Figure 5.**
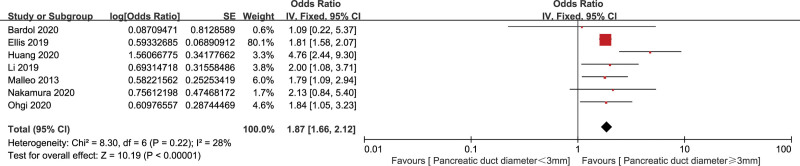
Forest plot of the relationship between CR-POPF and pancreatic duct diameter (3 mm). CR-POPF = clinically relevant postoperative pancreatic fistula.

Seven studies^[16,27,29,32,33,38,39]^ analyzed the relationship of pancreatic duct diameter <3 mm and CR-POPF, involving 12,325 patients. The fixed effects model was used for data synthesis because of a low heterogeneity (*I*^2^ = 28%) among the 7 studies. The pooled data showed that pancreatic duct diameter <3 mm was a significant risk factor of CR-POPF (OR = 1.87, 95% CI: 1.66–2.12, *P* < .001) (Fig. 5).

#### 3.2.4. Pancreatic texture

**Figure 6.**
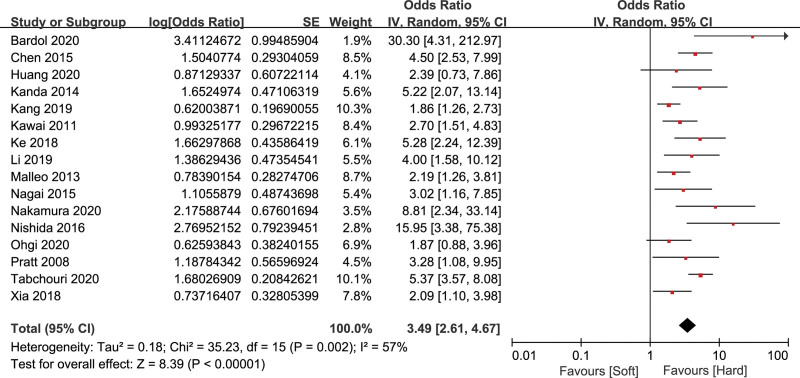
Forest plot of the relationship between CR-POPF and pancreatic texture. CR-POPF = clinically relevant postoperative pancreatic fistula.

Sixteen studies^[13,14,16–18,20,21,25,26,28,29,32–34,38,39]^ evaluated the relationship of pancreatic texture and CR-POPF, including 8203 patients. There was a high heterogeneity (*I*^2^ = 57%), so the random effects model was adopted. The result indicated that soft pancreatic texture was a significant risk factor of CR-POPF (OR = 3.49, 95% CI: 2.61–4.67, *P* < .001) (Fig. 6).

#### 3.2.5. Pathology type

**Figure 7.**
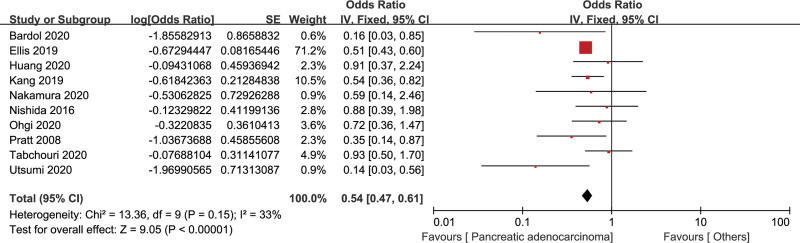
Forest plot of the relationship between CR-POPF and pathology type. CR-POPF = clinically relevant postoperative pancreatic fistula.

The relationship of pathology type and CR-POPF was assessed by 10 studies with 14,487 patients.^[13,21,27,28,32–35,38,39]^ There was a low heterogeneity (*I*^2^ = 33%), and the fixed effects model was used. The pooled data indicated that compared with other pathology types, pancreatic adenocarcinoma was a significant protective factor of CR-POPF (OR = 0.54, 95% CI: 0.47–0.61, *P* < .001) (Fig. 7).

#### 3.2.6. Diabetes mellitus

**Figure 8.**
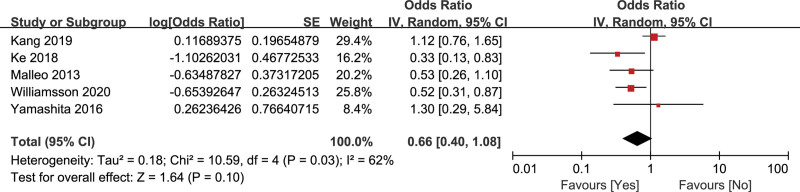
Forest plot of the relationship between CR-POPF and diabetes mellitus. CR-POPF = clinically relevant postoperative pancreatic fistula.

Five studies^[16,22,26,28,36]^ evaluated the relationship of diabetes mellitus and CR-POPF, containing 5247 patients. The random effects model was adopted because of a high heterogeneity (*I*^2^ = 62%). The result showed that diabetes mellitus could reduce the incidence of CR-POPF, but was not statistically significant (OR = 0.66, 95% CI: 0.40–1.08, *P* = .10) (Fig. 8).

#### 3.2.7. Blood transfusion

**Figure 9.**
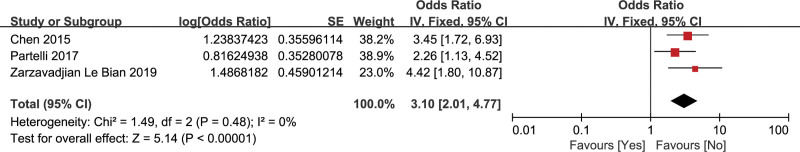
Forest plot of the relationship between CR-POPF and blood transfusion. CR-POPF = clinically relevant postoperative pancreatic fistula.

Three studies^[18,24,30]^ evaluated the relationship of blood transfusion and CR-POPF, including 1654 patients. The fixed effects model was used for data synthesis because no heterogeneity (*I*^2^ = 0%). The pooled data indicated that blood transfusion was a significant risk factor of CR-POPF (OR = 3.10, 95% CI: 2.01–4.77, *P* < .001) (Fig. 9).

#### 3.2.8. Vascular resection

**Figure 10.**
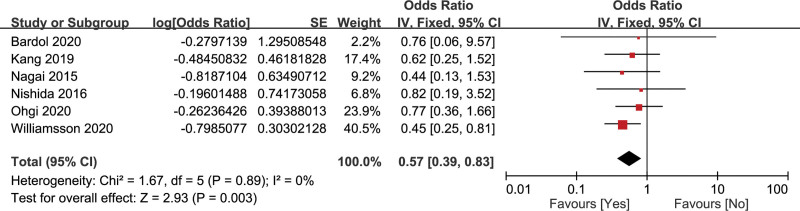
Forest plot of the relationship between CR-POPF and vascular resection. CR-POPF = clinically relevant postoperative pancreatic fistula.

The association of vascular resection and CR-POPF was assessed by 6 studies with 5462 patients.^[20,21,28,32,33,36]^ There was no heterogeneity (*I*^2^ = 0%), so the fixed-effects model was adopted. The result showed that intraoperative vascular resection was a significant protective factor of CR-POPF (OR = 0.57, 95% CI: 0.39–0.83, *P* = .003) (Fig. 10).

#### 3.2.9. Preoperative chemoradiotherapy

**Figure 11.**
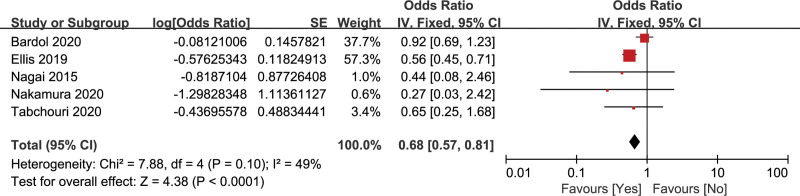
Forest plot of the relationship between CR-POPF and preoperative chemoradiotherapy. CR-POPF = clinically relevant postoperative pancreatic fistula.

The relationship of preoperative chemoradiotherapy and CR-POPF was assessed by 5 studies with 11,128 patients.^[20,27,32,34,39]^ There was a low heterogeneity (*I*^2^ = 49%), so the fixed effects model was adopted. The pooled data showed that preoperative chemoradiotherapy was a significant protective factor of CR-POPF (OR = 0.68, 95% CI: 0.57–0.81, *P* < .001) (Fig. 11).

### 3.3. Sensitivity analysis

There was a high heterogeneity in the analysis of BMI, pancreatic texture, and diabetes mellitus, so further sensitivity analysis was conducted. The effects of BMI and pancreatic texture on CR-POPF did not change by converting the effects model (Supplementary Figures 1 and 2, http://links.lww.com/MD/G805). Diabetes mellitus went from not statistically significant to a significant protective factor of CR-POPF by converting the random-effects model to the fixed effects model (Supplementary Figure 3, http://links.lww.com/MD/G805). Furthermore, the sources of heterogeneity were identified by excluding 1 study at a time. The heterogeneity decreased from *I*^2^ = 62% to *I*^2^ = 0% by excluding the study by Kang et al^[28]^ in the analysis of diabetes mellitus. The result after excluding the sources of heterogeneity showed that diabetes mellitus was a significant protective factor of CR-POPF (OR = 0.51, 95% CI: 0.35–0.74, *P* < .001) (Supplementary Figure 4, http://links.lww.com/MD/G805).

### 3.4. Publication bias analysis

**Figure 12.**
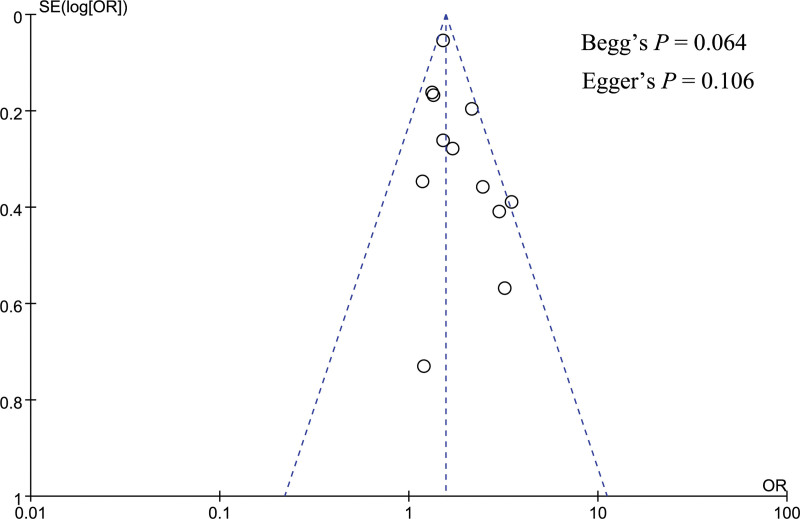
Funnel plot showed no significant publication bias in the analysis of gender.

**Figure 13.**
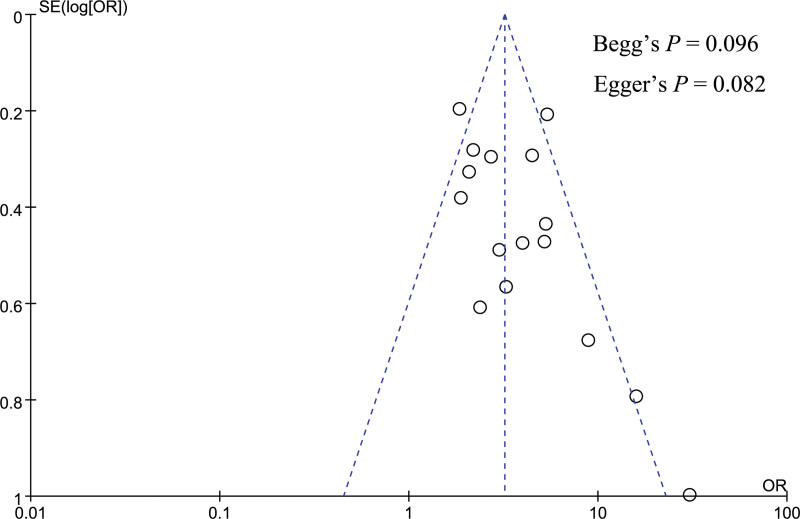
Funnel plot showed no significant publication bias in the analysis of pancreatic texture.

**Figure 14.**
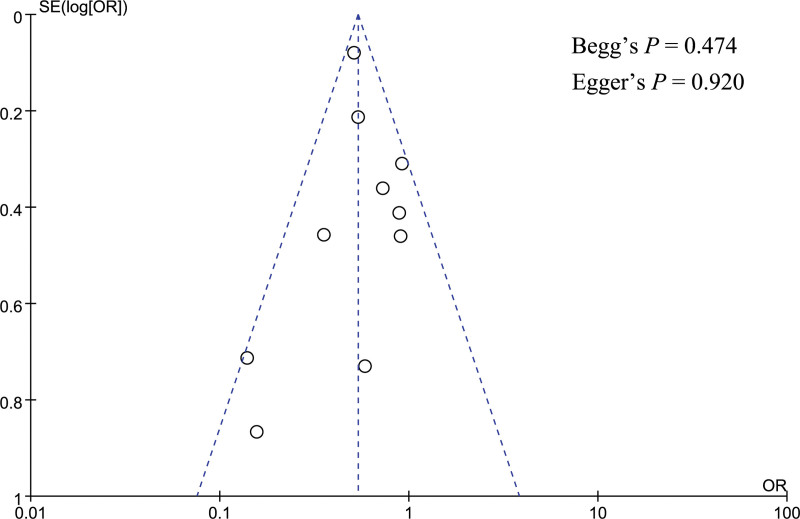
Funnel plot showed no significant publication bias in the analysis of pathology type.

Publication bias was performed for gender, pancreatic texture, and pathology type. A funnel plot was utilized to visually assess the publication bias, and the funnel plot looked symmetrical in the analysis of gender, pancreatic texture, and pathology type (Figs. 12–14). Besides, Begg and Egger tests were utilized to quantitatively assess publication bias, and the result showed no significant publication bias for gender (Begg test *P* = .064, Egger test *P* = .106), pancreatic texture (Begg test *P* = .096, Egger test *P* = .082), and pathology type (Begg test *P* = .474, Egger test *P* = .920).

## 4. Discussion

CR-POPF is a very common and troublesome complication after PD and is also associated with other complications such as postoperative bleeding, abdominal infection, and delayed gastric emptying. Therefore, it has an essential significance to prevent the occurrence of CR-POPF after PD. Identifying the risk factors of CR-POPF can provide a basis for the prevention and management of CR-POPF. The current meta-analysis showed males, BMI >25 kg/m^2^, pancreatic duct diameter <3 mm, soft pancreatic texture, and blood transfusion were significant risk factors of CR-POPF. Pancreatic adenocarcinoma, vascular resection, and preoperative chemoradiotherapy were significant protective factors of CR-POPF. Diabetes mellitus appears to reduce the incidence of CR-POPF, but more studies are needed to determine whether it is statistically significant.

As we know, BMI is a measure of overweight and obesity, and increased BMI is associated with perioperative adverse events such as longer operation time and postoperative incision infection.^[37]^ Our meta-analysis indicated that BMI >25 kg/m^2^ could significantly increase the risk of CR-POPF after PD (OR = 1.98, 95% CI: 1.23–3.18, *P* < .001). The study by Ellis et al^[27]^ demonstrated that compared with BMI <25 kg/m^2^, higher BMI could increase the risk of CR-POPF (OR = 1.40 for BMI = 25–30 kg/m^2^, OR = 1.97 for BMI >30 kg/m^2^). Increased BMI is associated with fatty pancreas and soft pancreatic remnant; studies^[40,41]^ showed that patients with POPF usually had significantly increased pancreatic fat and decreased pancreatic fibrosis.

Pancreatic duct diameter <3 mm and soft pancreatic texture were generally recognized risk factors of CR-POPF. The current meta-analysis showed that pancreatic duct diameter <3 mm could increase the risk by 1.87 times, and soft pancreatic texture could increase the risk of CR-POPF by 3.49 times. A smaller pancreatic duct diameter usually means a preserved exocrine function and more challenges for the reconstruction of the anastomosis, all of these can increase the risk of CR-POPF. Akamatsu et al^[42]^ found that POPF was associated with the ratio of main pancreatic duct diameter to pancreatic body (MPD index), and compared with MPD index ≥0.4, MPD index <0.4 was a significant risk factor (OR = 7.3 for MPD index = 0.2–0.4; OR = 50 for MPD index <0.2). There are several reasons why soft pancreatic texture can increase the risk of CR-POPF: the soft pancreas usually has a larger pancreatic body and a smaller pancreatic duct diameter: the soft pancreas generally has a smaller fibrosis ratio and a larger lobular ratio, which is associated with increased exocrine function; soft pancreas is more likely to be damaged by intraoperative dissection, suturing, and knotting; soft pancreas is more difficult to rebuild the anastomosis.^[25,26,42,43]^

The current meta-analysis showed pancreatic adenocarcinoma was a significant protective factor of CR-POPF (OR = 0.54, 95% CI: 0.47–0.61, *P* < .001). Besides, studies^[13,44]^ showed that chronic pancreatitis was also a protective factor of POPF. Nonpancreatic adenocarcinomas such as biliary, periampullary, and neuroendocrine tumors were usually characterized by soft and nonfibrous pancreas. However, pancreatic adenocarcinoma and chronic pancreatitis generally had a hard fibrotic pancreas and poor exocrine function, which can decrease the risk of CR-POPF.^[13,44,45]^

Diabetes mellitus is generally associated with postoperative adverse events. However, the effect of diabetes mellitus on CR-POPF remains controversial. Mathur et al^[41]^ found that patients with diabetes mellitus had more fibrosis and less fat in their pancreas and had less incidence of POPF compared with those without diabetes mellitus. Our meta-analysis showed that diabetes mellitus could reduce the incidence of CR-POPF, but was not statistically significant (OR = 0.66, 95% CI: 0.40–1.08, *P* = .10). Five studies were included in the analysis of diabetes mellitus, of which 2 studies showed diabetes mellitus was a significant protective factor of CR-POPF, and 3 studies showed no significant association between diabetes mellitus and CR-POPF. However, the analysis of diabetes mellitus had a high heterogeneity, and a further sensitivity analysis was performed. The result after sensitivity analysis showed that diabetes mellitus could significantly decrease the risk of CR-POPF. Therefore, further studies are necessary to identify the relationship between diabetes mellitus and CR-POPF. The effect of diabetes mellitus on CR-POPF may be related to its types and onset time.

Blood transfusion was a significant risk factor for CR-POPF in this meta-analysis. The reason that blood transfusion could increase the risk of CR-POPF was related to a large amount of intraoperative blood loss. Studies^[13,14]^ showed that intraoperative blood loss ≥1000 mL could significantly increase the risk of CR-POPF. Chen et al^[18]^ found that intraoperative blood loss ≥800 mL and blood transfusion were associated with an increased risk of CR-POPF (OR = 3.45, *P* < .001). Therefore, surgeons should minimize intraoperative bleeding in order to reduce the risk of CR-POPF.

The current meta-analysis showed that intraoperative vascular resection was a protective factor of CR-POPF after PD. The study by Williamsson et al^[36]^ showed that vascular resection was associated with neoadjuvant therapy and a large tumor, and a large tumor that required vascular resection was more likely to block the pancreatic duct and increase the stiffness of the pancreas. Besides, a recent study by Zettervall et al^[46]^ showed that vascular reconstruction in PD could successfully achieve microscopically negative margins (R0) resection, and venous reconstruction was safe without increasing the 30-day morbidity and mortality, but arterial reconstruction significantly increased 30-day morbidity and mortality.

Neoadjuvant therapy that preoperative chemoradiotherapy for malignant tumors has increased significantly over the last decades. A study^[47]^ showed preoperative chemoradiotherapy was an independent risk factor for symptomatic anastomotic leakage following anterior resection of the rectum. However, preoperative chemoradiotherapy appears to be a protective factor for CR-POPF after PD. And our meta-analysis showed that preoperative chemoradiotherapy could significantly decrease the risk of CR-POPF (OR = 0.68, 95% CI: 0.57–0.81, *P* < .001). Preoperative chemoradiotherapy was associated with increased pancreatic fibrosis and decreased exocrine function, which can decrease the risk of CR-POPF.^[27,34]^

## 5. Strengths and limitations

The current meta-analysis assessed the effect of different factors on CR-POPF after PD, and grade A PF with no clinical impact was excluded. This can help clinicians better judge the risk of CR-POPF after PD, thus providing a basis for the prevention and management of CR-POPF. For high-risk patients, we should be vigilant and take effective measures to prevent the occurrence of CR-POPF. Vuorela et al^[48]^ found that pasireotide could reduce the occurrence of CR-POPF in high-risk patients with small pancreatic duct, soft pancreatic texture, biliary, or neuroendocrine tumors. The study by Lyonell et al^[49]^ showed that duct-to-mucosa or invagination techniques were not statistically associated with CR-POPF for patients with mean size of pancreatic duct, but duct-to-mucosa was preferable for patients with pancreatic duct diameter of >6 mm.

There were also some limitations in this meta-analysis. First, most included studies were retrospective, and more prospective studies were necessary for further assessing the risk factors of CR-POPF. Second, the association between some factors and CR-POPF were not discussed in this meta-analysis because the cutoff value of some factors was inconsistent in different studies or some factors were reported in <3 studies, such as age, hypoproteinemia, surgical techniques, etc.

## 6. Conclusion

The current meta-analysis indicated that males, BMI >25 kg/m^2^, pancreatic duct diameter <3 mm, soft pancreatic texture, and blood transfusion were significant risk factors of CR-POPF. Pancreatic adenocarcinoma, vascular resection, and preoperative chemoradiotherapy were significant protective factors of CR-POPF. Diabetes mellitus appeared to decrease the incidence of CR-POPF, but this still needs further studies to assess. Effective interventions targeting the above-mentioned risk factors should be investigated in future studies for decreasing the occurrence of CR-POPF.

## Author contributions

Conceptualization: Biao Zhang and Dong Shang.

Data curation: Biao Zhang and Qihang Yuan.

Funding acquisition: Dong Shang.

Investigation: Shuang Li and Zhaohui Xu.

Methodology: Xu Chen and Lunxu Li.

Project administration: Dong Shang.

Supervision: Biao Zhang.

Visualization: Biao Zhang and Qihang Yuan.

Writing – original draft: Biao Zhang, Qihang Yuan, Shuang Li, and Zhaohui Xu.

Writing – review and editing: Dong Shang.

## Supplementary Material


