# Supplementary figures and images for: Risk factors of clinically relevant postoperative pancreatic fistula after pancreaticoduodenectomy: A systematic review and meta-analysis
Source: Medicine (Baltimore). 2022 Jun 30;101(26):e29757. doi: 10.1097/MD.0000000000029757 (PMC9239615; doi:10.1097/MD.0000000000029757)

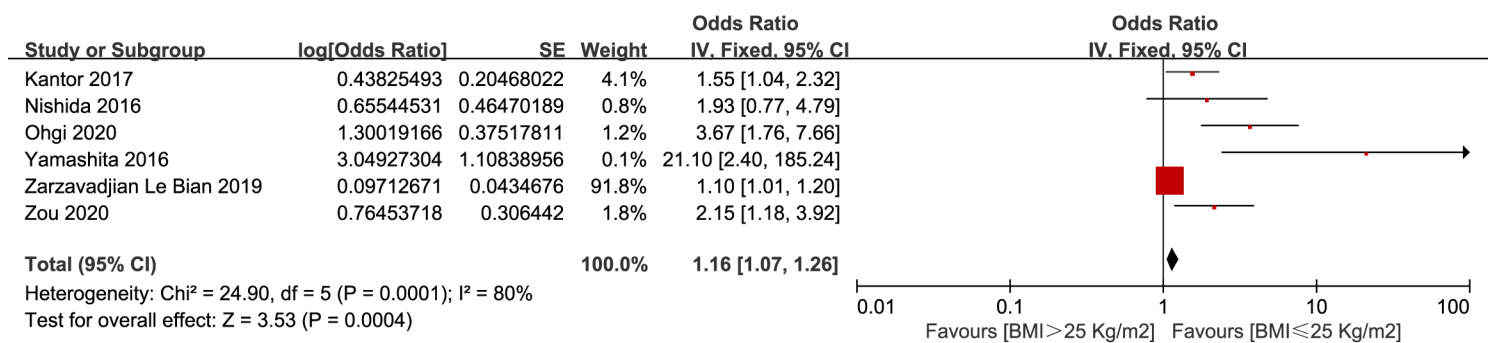

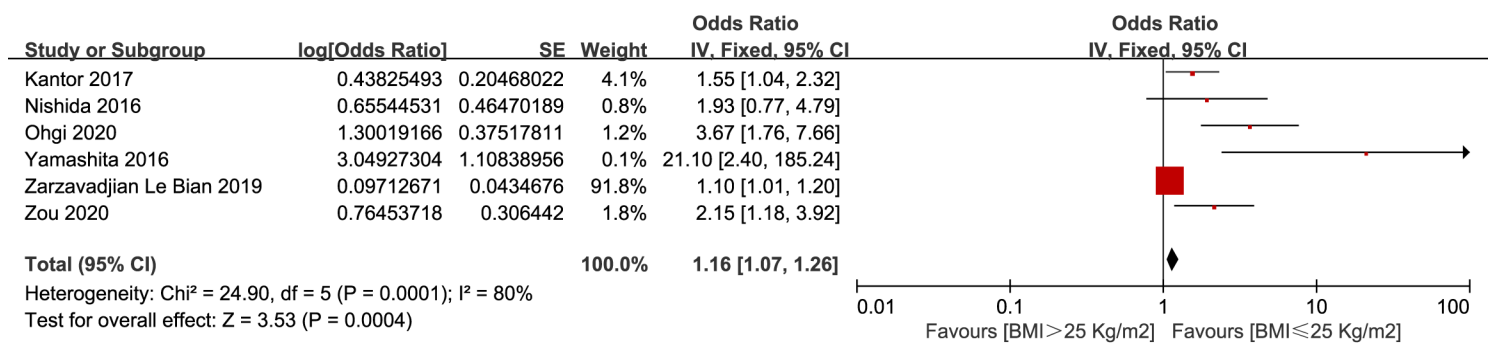

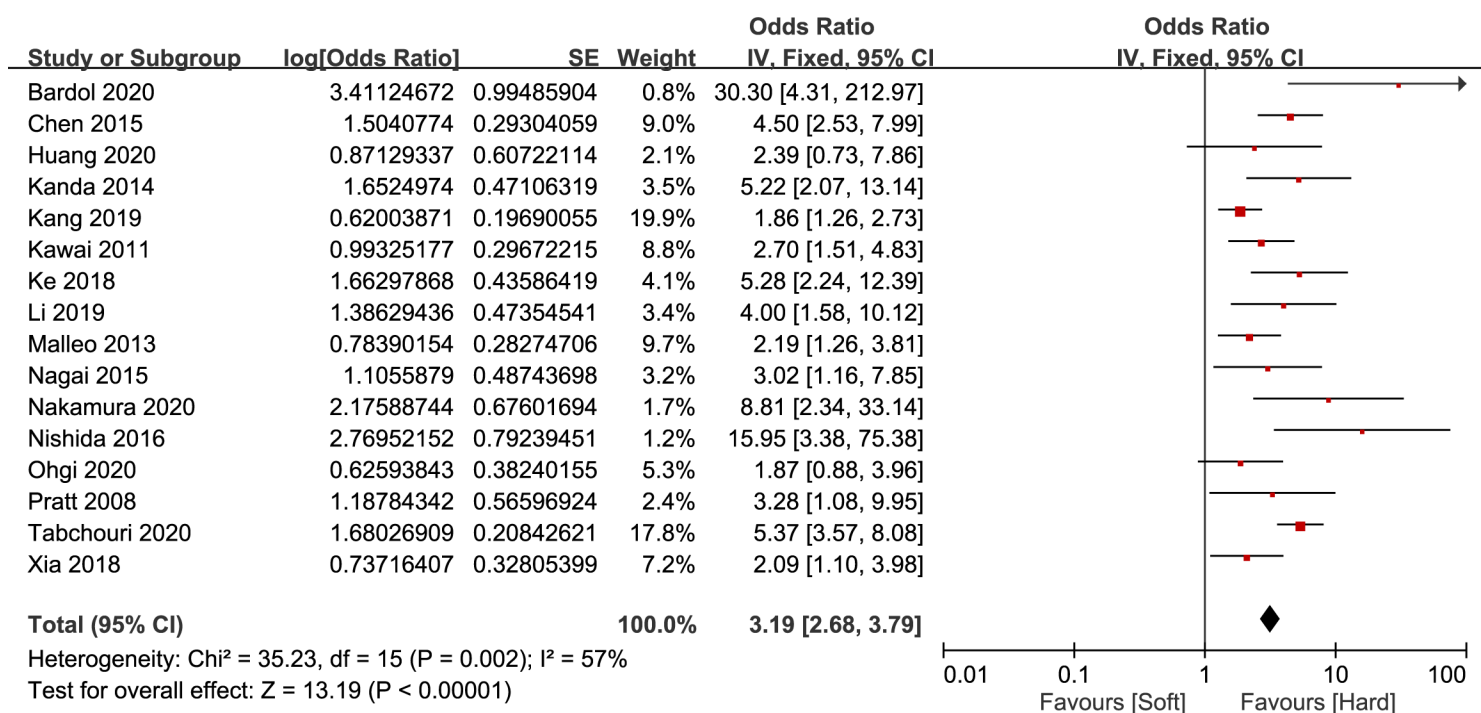

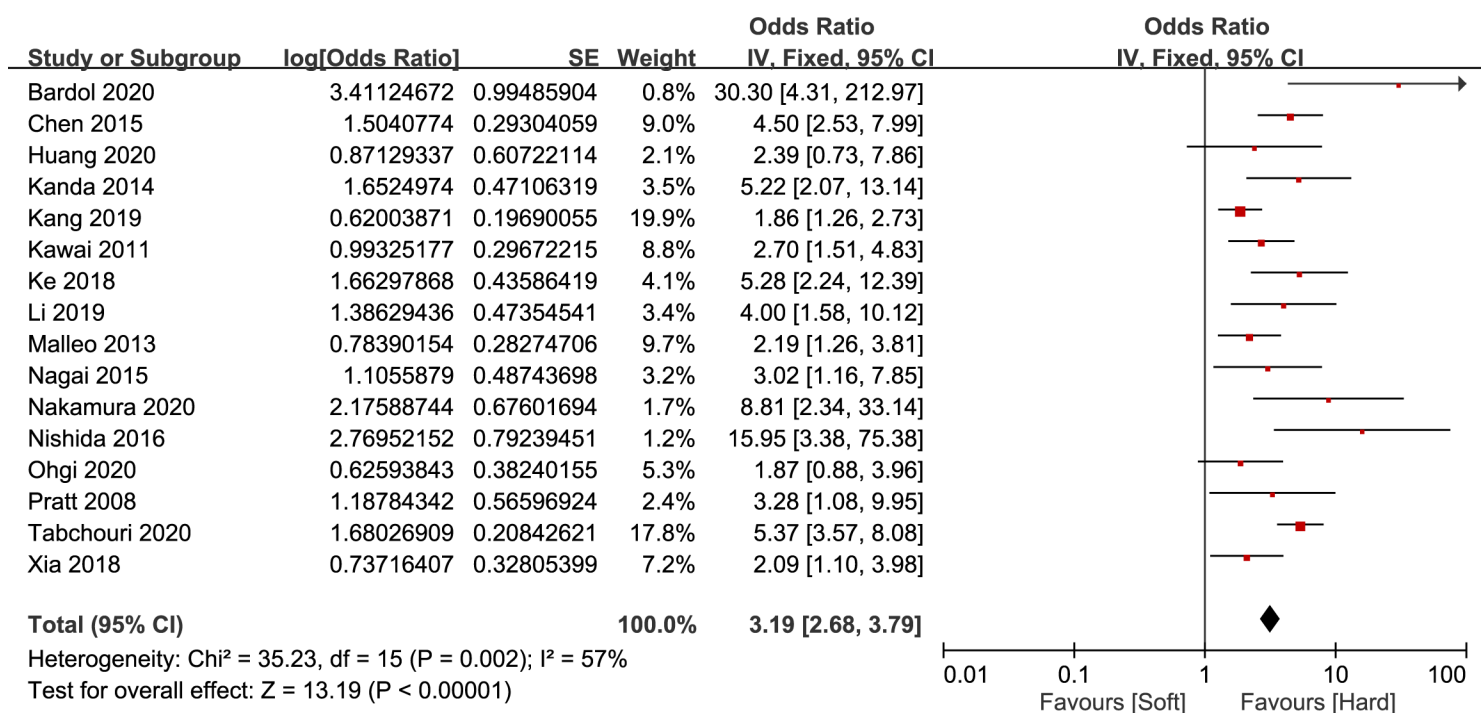

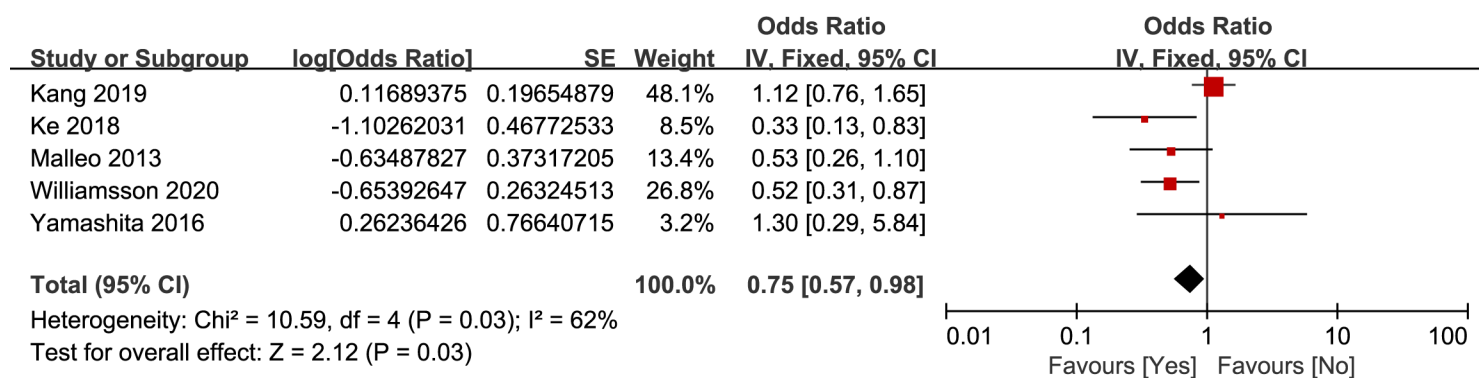

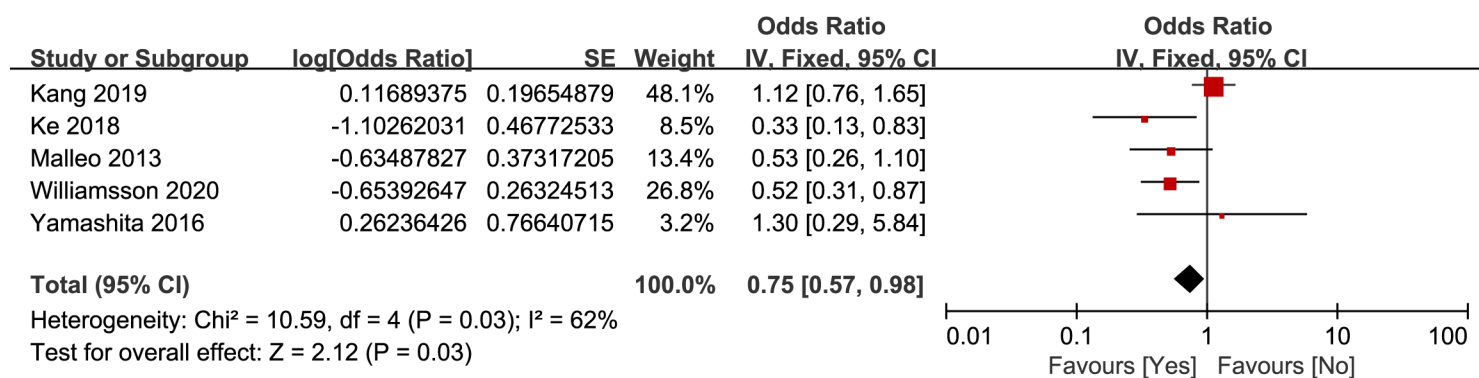

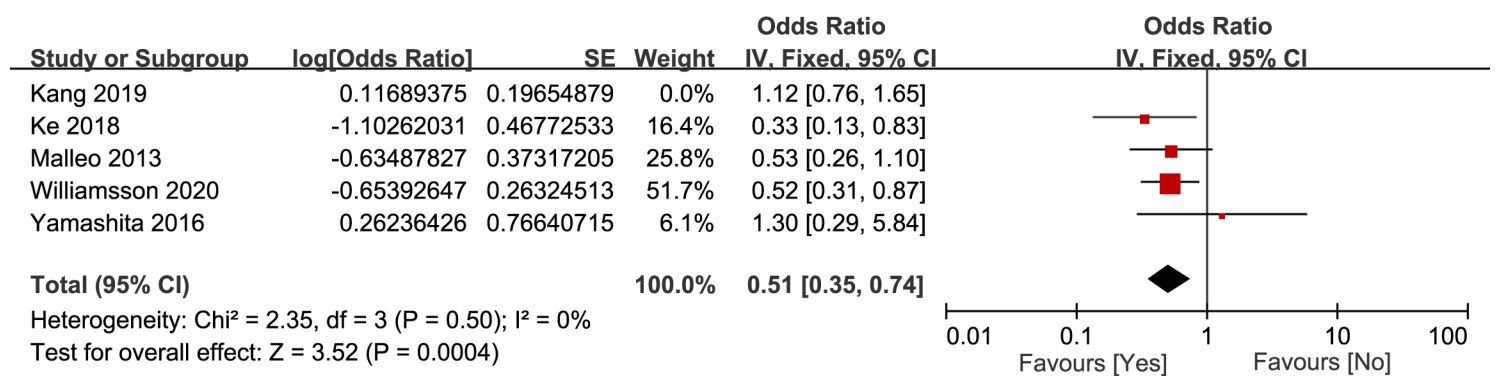



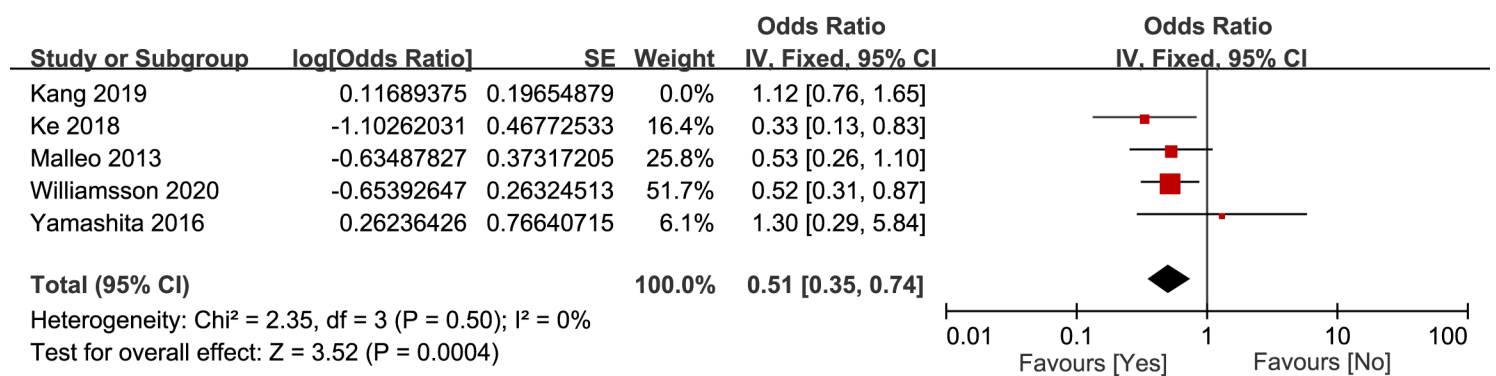

Supplement: Supplementary file 1 [file medi-101-e29757-s001.pdf]
